# Characterization of Oviduct Lining, with Emphasis on the Sperm Storage Tubule Region (Uterovaginal Junction), Correlated with Fertility in Mature and Old Thai Native Hens

**DOI:** 10.3390/ani11123446

**Published:** 2021-12-03

**Authors:** Theerapat Kheawkanha, Wuttigrai Boonkum, Thevin Vongpralub, Vibuntita Chankitisakul

**Affiliations:** 1 Department of Animal Science, Faculty of Agriculture, Khon Kaen University, Khon Kaen 40002, Thailand; k.theerapat@kkumail.com (T.K.); wuttbo@kku.ac.th (W.B.); vthevi@kku.ac.th (T.V.); 2 Network Center for Animal Breeding and Omics Research, Faculty of Agricultural, Khon Kaen University, Khon Kaen 40002, Thailand

**Keywords:** aged hen, SST, Thai indigenous chicken

## Abstract

**Simple Summary:**

Although a reduction in the number of sperm in the sperm storage tubule (SST) regions in older hens was hypothesized to relate to fertility, no comparison has been made to date on the effect of age (mature and old) on reproduction, the morphology of the sperm storage reservoir, the resident sperm in the UVJ, and the fertility duration in chickens. The present study indicated the morphological characteristics of reproductive organs did not differ between mature and old hens. The numbers of ovarian follicles were greater in mature hens. Significant differences in the sperm storage reservoir, such as the inner and outer diameter of the SSTs and epithelium height, were demonstrated. The number of resident sperm cells that were related to fertility periods was greater in mature hens than in old hens.

**Abstract:**

The effect of age on fertility was investigated in Thai native chickens. The objective of this study was to determine the effects of age (mature and old) on the morphological characteristics of the reproductive organs and the histological characteristics of the uterovaginal junction (UVJ) tissues, resident sperm in the UVJ, and fertility duration in Thai native hens. We found no differences in the morphological characteristics of the reproductive organs, except for the number of follicles and the sizes of the fifth large yellow follicle in mature hens, which were greater than those in old hens (*p* < 0.05). The diameter of the sperm storage tubules (SSTs) epithelium was larger in old hens than in mature hens (*p* < 0.05), whereas the epithelium height was lower in old hens (*p* < 0.05). The number of sperm in the SSTs was greater in mature hens compared with old hens (*p* < 0.05). Mature hens showed a higher fertility rate than old hens. Our results suggest that, in old hens, the function of the SSTs is impaired, and sperm cannot be retained. Such a deterioration of the SSTs may be one of the factors involved in the decline in fertility.

## 1. Introduction

Flock fertility in chickens depends on both the males’ and females’ reproductive status, including factors such as sperm quality, behavior, egg quality, and animal age. Egg production begins when the hen reaches 18–22 weeks, depending on the breed and season [1]. Fertility usually increases from a low of 65–75% at the start of laying and peaks at 95% at 35–37 weeks of age, followed by a decline; the most significant drop in fertility occurs at 45–54 weeks of age [2,3,4,5]. More frequent mating is needed to sustain high fertility after 40 weeks of age [6]. It is apparent that sperm penetration is greatly reduced, dependent upon the age of the flock [7]. However, the physiology of sperm from older males appears to be relatively unaffected in terms of fertilization and penetration abilities [7,8]. One major contributing factor to decreased sperm penetration is more pronounced in aged females due to the hens’ physiological status. The conditions that cause low sperm numbers or single sperm activity at the site of fertilization could be associated with reduced fertility.

Sperm storage within the female reproductive tract is important for maintaining fertility, specifically in animal species with asynchronous copulation and ovulation [9]. After copulation, either via natural mating or artificial insemination, in female poultry species, i.e., hens, turkey, and birds, sperm is transported through the cloaca to the junction of the uterus and the vagina (uterovaginal junction (UVJ)) of the oviduct [10,11]. In the UVJ, sperm enter the tubular invagination sites of the surface epithelium of the mucosa, collectively called sperm storage tubules (SSTs), where they are stored for longer periods, depending on the species, and retain their fertilizing capacity [12,13]. In general, the sperm storage tubules of hens are full for 24–48 h after insemination [14,15]; a reduction in the number of sperm in older hens was hypothesized to relate to a lack of sperm storage [7]. However, there has never been a report to date that compared the correlation of SST characteristics and fertilization capacity in mature and old chickens. The objectives of this study were to investigate the effects of age (mature and old) on the differences in reproductive organs, the morphological characteristics of the sperm storage reservoir, resident sperm in the UVJ, and fertility duration in Thai native hens. These results might be helpful to explore the physiological action of the SSTs of hens at different ages, with the prospect of further improving fertility management in flocks.

## 2. Materials and Methods

### 2.1. Animals, Housing, and Feeding

In total, 76 virgin Thai native hens were divided into the following groups: 38 hens that were 35–39 weeks old (the peak production duration; mature group), 38 hens that were 73–75 weeks old (the end of the production period; old group), and 12 roosters; the animals were raised in individual cages with standard feeding and management under natural conditions. The animals were randomly assigned to each experiment, except for twenty hens in the old group for Experiment 3, who were selected from the top 20% of egg producers of the flock to gain more egg production for the fertility test.

### 2.2. Experimental Design

#### 2.2.1. Experiment 1: Effect of Age on Morphological Characteristics of Reproductive Organs and Histological Characteristics of UVJ Tissues

To characterize the reproductive organs and the UVJ histology of the SSTs for mature and old hens, the hens (*n* = 6 per group) were sacrificed using decapitation to dissect the reproductive organs. The reproductive organs, namely, the ovary and oviduct, were weighed and measured, and the number of ovarian follicles was determined. The UVJ tissues containing the SSTs were prepared and stained with hematoxylin and eosin for histological examination.

#### 2.2.2. Experiment 2: Effects of Ages on Resident Sperm in the Uterovaginal Junction after Artificial Insemination (AI) 

To assess the resident sperm after AI, the hens (*n* = 12 per group) were artificially inseminated. Hens were sacrificed using decapitation 24 h following AI to dissect the UVJ tissues containing the SSTs under a stereomicroscope. Half of the hens in each group were examined in terms of sperm recovery by flushing the resident sperm and the others were examined regarding the percentage of SSTs containing sperm by tissue staining using hematoxylin and eosin to determine the number of SSTs containing sperm.

#### 2.2.3. Experiment 3: Effects of Age on Fertility

To examine fertility, the hens in each group (*n* = 20 per group per time) were artificially inseminated twice, with a 15-day interval (starting at 35 and 73 weeks of age in the mature and the old groups, respectively). Diluted fresh semen at a final concentration of 150 × 10^6^ sperm/dose were used. Eggs were collected for 14 days during days 2–15 after insemination, and fertility was determined by candling eggs on day 7 of incubation. 

### 2.3. Morphological Characteristics of Reproductive Organs and Histological Characteristics of UVJ Tissues

After weighing and sacrificing using decapitation, the entire reproductive system (ovary to vent) was carefully removed. The weights (g) of the ovary and the oviduct (emptied of contents) were measured using a weighing balance (Adam NBL214i, Adan Equipment Co. Ltd., Jing An, Shanghai, China) and photographed to measure the oviductal length. The number and size of the follicles were determined by using image analysis software (ImageJ software, National Institutes of Health (NIH), Bethesda, Maryland, USA); the different follicles were classified into four categories, as described by Ebeid et al. [16]: small white follicles (SWFs; 1–3 mm), large white follicles (LWFs; 3–5 mm), small yellow follicles (SYFs; 5–10 mm), large yellow follicles (LYFs; >10 mm), and total follicles (TFs). The top five largest yellow follicles in each group were compared.

The histological examination of UVJ tissues was carried out as described previously [17]. Briefly, the UVJ tissues were identified and dissected under a stereomicroscope, where they appeared as a distinct band of thin and convoluted folds [11]. Tissues were immediately fixed in 10% formalin in PBS for 24 h and embedded in paraffin. Then, UVJ longitudinal sections with a thickness of 6 µm were cut and stained with hematoxylin and eosin on glass slides, followed by examination under a digital pathology scanner with computer-assisted software for image analysis (Aperio ImageScope, Leica Biosystems, Buffalo Grove, IL, USA). The characteristics of the UVJ sections in terms of the number of folds, fold length, epithelium height, and outer and inner diameters of the SSTs were evaluated. At least five SST structures were analyzed on each slide, and four to six replications of the UVJ mucosa fold tissue samples were evaluated.

### 2.4. Semen Collection, Evaluation, and AI

Semen was collected using the dorso-abdominal massage method [18]. Semen from individual roosters was collected in a 1.5 mL microtube containing 0.1 mL of IGGKph diluent, which was composed of 0.14 g potassium citrate, 1.40 g sodium glutamate, 0.21 g sodium dihydrogen phosphate, 0.02 g protamine sulfate, 0.98 g anhydrous sodium hydrogen phosphate, 0.70 g glucose, 0.02 g fructose, 0.70 g inositol, and 0.10 polyvinylpyrrolidone in 100 mL distilled water [19]. Within 15 min after semen collection, semen samples were examined under a microscope and determined based on the following criteria: mass motility score ≥ 4 (score range 0–5, phase contrast microscope ×40), sperm concentration ≥ 3 × 10^9^ sperm/mL (hemocytometer counting method), and sperm viability and morphology of sperm ≥ 85% (eosin-nigrosine straining method, phase contrast microscope ×100). After semen evaluation, the semen samples that passed the evaluation criteria were pooled to increase the semen volume and reduce the bias. Then, semen was re-diluted with IGGKph at a final concentration of 150 × 10^6^ sperm/dose (0.2 mL) diluent for insemination. 

Artificial insemination was performed using a tuberculin syringe containing diluted semen inserted into the vagina (approximately 4 cm). Insemination was performed between 1.00 and 3.00 p.m. [20].

### 2.5. Sperm Recovery

Sperm recovery of the UVJ section was examined following the modified method described by Brillard and Bakst [21]. Briefly, the hens were sacrificed using decapitation to dissect the reproductive organs. The proximal end of the uterine pouch and the distal end of the vagina were clamped, and the vagina was stripped of connective tissue until it was straight. Each clamped segment was injected with 3.5 mL Dulbecco’s Modified Eagle Medium (DMEM) (Sigma, St. Louis, MO, USA) and gently massaged for 30 s to disseminate the wash solution within the mucosal folds. The washing fluid was collected, and the volume of recovered fluid was recorded. The number of sperm was determined by a hemocytometer under phase-contrast microscopy (Olympus CH30, Tokyo, Japan) at ×100 magnification. Sperm heads were easily distinguished from cell debris because they were consistently packed, slightly arced, filiform-shaped, and approximately 0.6 µm in diameter. Six replicates were counted per washing. The total number of spermatozoa in a sample was calculated according to the following formula: average hemocytometer count × pipette dilution rate (20 µL) × volume of flushing × 10^4^.

After washing, the UVJ tissues were subsequently sliced lengthwise, and their mucosae were exposed to collect the total number of spermatozoa that were living in the SSTs. By gently scraping the surface folds and lamina propria from the muscularis mucosae in the vagina, approximately 8 cm from the distal boundary of the uterus, the surface folds and lamina propria were separated from the muscularis mucosa. The mucosa containing the SSTs was isolated, minced in Dulbecco’s Modified Eagle Medium (DMEM), and dissociated with a 2.5% (*v*/*v*) solution of collagenase (Type XI from Sigma) that was diluted in Hank balanced salt solution (HBSS). Digestion was performed using 500 µL diluted collagenase for 400 mg tissue, which was then re-diluted 1:3 (*v*/*v*) in HBSS. Digestion was carried out in a water bath at 37 °C for 45 min with shaking [22]. Spermatozoa in the dispersed SST-containing mucosa were counted with a hemocytometer under phase-contrast microscopy (Olympus CH30, Tokyo, Japan) at ×100 magnification. Six replicates were counted per washing, and the total number of spermatozoa in a sample was calculated according to the following formula: average hemocytometer count × dilution rate (1:3) × volume of sample × 10^4^. The total numbers of sperm from two parts were combined and to represent the sperm recovery.

### 2.6. Percentages of SSTs Containing Sperm 

The sperm content in the UVJ section was used to quantify the sperm storage capacity. The UVJ tissue examination was carried out as mentioned above. Three sections that were used for the classification of the SSTs with sperm were examined under a digital pathology scanner with computer-assisted software for image analysis (Aperio ImageScope, Leica Biosystems, Buffalo Grove, IL, USA). Six folds of the UVJ tissue from each SST were evaluated, and the percentage of SSTs containing spermatozoa was calculated by dividing the number of SSTs with sperm by the number of SSTs from each UVJ fold.

### 2.7. Fertility Test 

Fertilizing ability was tested using single intravaginal insemination of the hens at day 0. Eggs were collected during days 2–15 after each insemination before incubation. The number of eggs that were laid was recorded. The percentage of hen-day egg production was calculated by dividing the number of eggs produced on daily basis by the number of birds available on the day. To determine the fertility, eggs were examined using candling on the 7th day of incubation. The fertility rate was calculated by dividing the number of fertilized eggs by the number of incubated eggs.

### 2.8. Statistical Analysis

Student’s *t*-test was used to determine the significant differences for all parameters (*p* < 0.05). The results were analyzed using the SAS 9.0 statistical software program (SAS Institute, Inc., Cary, NC, USA) by using “PROC TTEST” to determine the differences between mature and older hens.

## 3. Results

### 3.1. Experiment 1: Effect of Age on Morphological Characteristics of Reproductive Organs and Histological Characteristics of UVJ Tissues

The mean (± SEM) values for the body weight, numbers of large yellow follicles, ovary weight, oviductal weight, and oviductal length of hens of different ages are shown in Table 1. The body weight of the mature chickens (1.8 ± 0.0 kg) was significantly lower than that of the old chickens (2.1 ± 0.1 kg) (*p* < 0.05). However, there were no statistically significant differences in the ovary weight, oviductal weight, and oviductal length between mature and old hens.

Based on the data in Table 2, the numbers of white and yellow follicles in terms of SWFs, LWFs, LYFs, and TF in mature hens were significantly higher than those in old hens (*p* < 0.05), whereas the SYF numbers were only slightly different (*p* = 0.10). Regarding the diameter of the top five largest yellow follicles, there were no significant differences, except for the fifth (F5) large yellow follicle in mature hens, which was larger than that of old hens (*p* < 0.05).

Table 3 shows the results of the image analysis for the SST structure in mature and old hens. The number of folds, fold length, and SSTs per fold did not differ between the groups (*p* > 0.05). The outer and inner epithelium diameters of the SSTs that were located throughout the entire region of the UVJ were larger in old hens (55.5 ± 8.9 µm and 27.5 ± 3.7 µm) than in mature hens (30.6 ± 5.8 µm and 6.9 ± 1.7 µm) (*p* < 0.05). In contrast, the epithelium height of the SSTs was lower in old hens (13.6 ± 2.6 µm) than in mature hens (16.3 ± 1.3 µm) within the whole region of the UVJ (*p* < 0.05).

**Figure 1:**
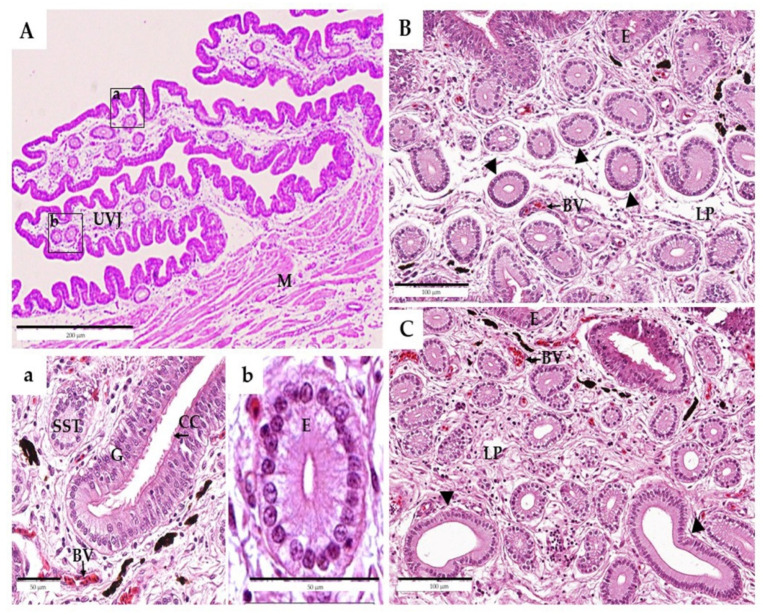
Histological examination of sperm storage tubules (SSTs) in the uterovaginal junction (UVJ) of non-artificially inseminated Thai native hens. Diagrammatic representation of the main sperm storage site in the UJV, consisting of folds of the epithelium (**A**). The UVJ epithelium cell was lined by a pseudostratified columnar type, with an abundance of SSTs in the lamina propria submucosa region (**a**). Each SST was lined with a columnar epithelium resting on a basal lamina and was situated close to blood vessels; it contained darkly stained basal nuclei with a lightly stained cytoplasm (**b**). In young hens, the arrow represents the examination of SSTs with a narrow luminal cavity and a single layer of tall epithelium cells (**B**). Old hens showed an inflated morphology with a wider lumen cavity and the SST cells were slightly flattened (**C**). BV—blood vessel, CC—ciliated cell, E—epithelium, G—goblet cell, LP—lamina propria, M—muscularis, S—stoma, and SST—sperm storage tubule. Scale bars: 50 µm (**a**,**b**), 100 µm (**B**,**C**), and 200 µm (**A**).

Generally, the UVJ is located at the cranial/anterior end of the vagina, which is also morphologically distinct from the vagina and the uterus inside and consists of round folds of the epithelium (Figure 1A). The epithelium at the UVJ was lined with pseudostratified columnar-type structures that were ciliated or non-ciliated with goblet and basal cells, respectively (Figure 1a). An abundance of SSTs was present in the lamina propria of the mucosal folds with a distinct lumen. Those SSTs were observed in the UVJ, and these tubules were mostly ranched, slightly coiled, and extended into the lamina propria from the bases of the mucosal folds. Each SST was lined with a columnar epithelium that rested on a basal lamina and was situated close to blood vessels. They contained darkly stained basal nuclei with lightly stained cytoplasm (Figure 1b).

In the lamina propria submucosa, differences in the SSTs between mature and old hens were observed (Figure 1B,C). The SSTs of both groups consisted of a single layer of simple columnar epithelium cells containing a round nucleus at the basal region. In mature hens, each SST was surrounded by a loose connective tissue stroma, and the luminal cavity was a narrow and apical surface of SST cells that could be moderately stained with eosin (Figure 1B). When compared to mature hens, the SSTs of the old hens showed an inflated morphology with a wider lumen cavity, and the SST cells were slightly flattened (Figure 1C).

### 3.2. Experiment 2: Effects of Age on Resident Sperm in the Uterovaginal Junction after Artificial Insemination (AI)

**Figure 2:**
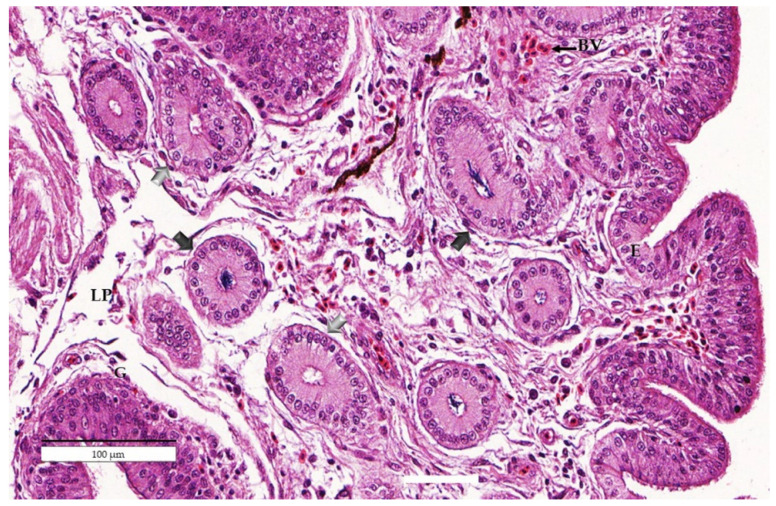
Transverse section of the uterovaginal junction (UVJ) fold of a mature Thai native hen after insemination for 24 h containing sperm storage tubules (SSTs) stained with hematoxylin and eosin (H and E). SSTs have the capacity to store different sperm: black arrows indicate sperm residing in the SST, while the gray arrow showed that the SSTs were not responsible for sperm retention. BV—blood vessel, E—epithelium, and LP—lamina propria. Scale bar: 100 µm.

Table 4 compares the sperm number recovery from the mucosa washing and the sperm contents in the UVJ between the two groups. The sperm number (×10^4^) that was recovered from mucosa washing was greater in mature hens (143.2 ± 14.0) compared with old hens (114.6 ± 17.1) (*p* = 0.03). The percentage of SSTs containing sperm in mature hens (26.8 ± 3.4%) was higher than that in old hens (6.8 ± 1.7%) (*p* < 0.01) (Figure 2).

### 3.3. Experiment 3: Effects of Age on Fertility

**Figure 3:**
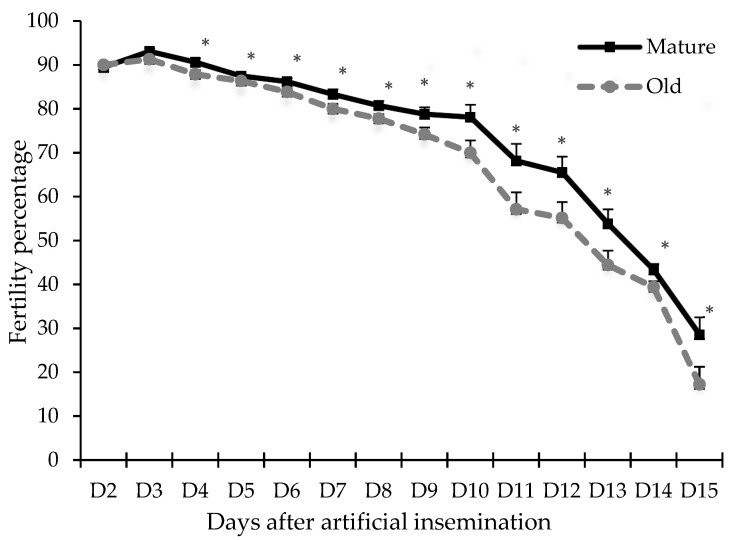
Fertility percentage (%) comparison between mature and old hens after a single artificial insemination with 150 × 10^6^ sperm (0.2 mL). Values are percentages of fertilized eggs per group and per day of collection; asterisks indicate significant differences at *p* < 0.05 (Student’s *t*-test).

The percentages of hen-day egg production in the present study were 75.5 ± 12.4 and 69.8 ± 13.6 in the mature and old hens, respectively. Figure 3 shows the fertility periods for the mature and old hens; the fertility rate of the mature group was higher than that of the old group from day 3 onward (*p* < 0.05).

## 4. Discussion

Flock fertility in old chickens depends on various factors, such as sperm storage within the uterovaginal junction of the oviduct. It was hypothesized that the decline in the fertility of old hens was due to a decreasing sperm storage capacity [23]. In the present study, we compared the effect of age (mature and old) on reproduction, the morphology of the sperm storage reservoir, resident sperm in the UVJ, and fertility duration in Thai native chickens. The morphological characteristics of the reproductive organs did not differ between mature and old hens. Meanwhile, the numbers of ovarian follicles were greater in mature hens. Significant differences in the sperm storage reservoir, such as the inner and outer diameters of the SSTs and epithelium height, were demonstrated. The number of resident sperm cells, which related to fertility periods, was greater in mature hens than in old hens.

We found significantly different body weights in old hens, which were greater than those of mature hens. This might be correlated with an increase in abdominal fat content in older hens compared with mature hens [24]. However, the reason the weights of the oviducts did not differ between groups might have been due to a similar feeding regimen [25]. In general, ovarian follicles can be divided into prehierarchical and hierarchical follicles (or preovulatory follicles). The prehierarchical follicles can be divided into SWFs, LWFs, SYFs, and LYFs, while the latter category is named F1–F5 according to the size of the follicle [16]. It was reported that the follicle-stimulating hormone is an essential factor that regulates all four stages of the prehierarchical follicles. Meanwhile, a high concentration of luteinizing hormone and progesterone might promote the development of prehierarchical follicles into hierarchical follicles and improve egg production in laying geese [26]. Similar to our results, the number of ovarian follicles in the prehierarchical and hierarchical follicles was more significant in mature hens (Table 2). This might result in the fact that mature hens have more egg production compared with aging hens. This agrees with the findings of Zakaria et al. [27], who reported an effect of aging on the ovarian follicular growth of layers aged from 20 to 92 weeks; the number of growing follicles increased from 20–32 weeks and declined from 44–92 weeks. Typically, egg production in commercial layers begins when the chickens reach maturity, depending on the breed and the season. Flock production rises sharply and reaches a peak 6–8 weeks later, followed by a gradual decline to about 65% after 12 months [1]. In Thai native chickens, our previous studies reported that the production peak occurred in the third month of the egg production period, after which, the monthly number of eggs decreased until the end of the production period [28,29]. Therefore, we inferred that the egg production performance was related not only to feeding management and environmental control [30,31] but also to follicular development.

The decreasing ability of SSTs to preserve sperm, which is caused by aging, is thought to be a major contributing factor to decreased fertility in aged hens. The reservoir of sperm within the SST tissues ensures that sperm are available between inseminations and ideally secures a sustained probability of fertilization [32]. Maximal filling of the SSTs occurs during the first 24–48 h after insemination and is essential for the series of fertilized eggs that typically follow a single insemination [14,15]. In the present study, the percentage of SSTs containing sperm at 24 h following AI in mature hens was higher than that in old hens (Table 4 and Figure 2), indicating that sperm entry into the SSTs was impeded in old hens; in other words, the older hens were not able to maintain sperm in their SSTs. The lower sperm capacity in the old hens was not due to the saturation of SSTs, as the percentage of SSTs with sperm was never full, even in the younger hens [15]. Currently, the mechanisms by which sperm could enter the SSTs are still largely unclear. However, the results of the hematoxylin and eosin staining of the UVJ epithelium and SST sections from both ages were not different (Figure 1). This agrees with the finding of Yang et al. [26], who reported that they did not find histologically characterized differences in UVJ and SSTs structure at three stages (30, 65, and 120 weeks of age) in White Leghorn hens. However, we found significant differences in the inner and outer diameters of the SSTs and the epithelium heights. The large inner diameter of the SSTs in old hens resulted in a wider lumen of the SSTs (Figure 1), which was associated with swelling of the SST structure, which might explain the declined sperm reservoir within the SSTs in old hens. This was also reported by Das et al. [17], who suggested the destruction of the SSTs, including swelling, in older hens. This phenomenon might explain why older hens with long-term use are more susceptible to inflammation reactions caused by AI, resulting in SST destruction [33,34]. However, our experiment could not explain this phenomenon, as these hens were virgins, and only eggs passed through their reproductive tracts. Another hypothesis of the cause of such swelling might be that the direct mechanical impact of the larger sizes and number of eggs in older hens was possibly associated with changes in the overall functionality of the reproductive tract, as described by Brillard [15].

Besides the more remarkable sperm contents in SSTs of mature hens, sperm could be stored in the SSTs of mature hens for a longer period compared to old hens, resulting in higher fertility in mature hens (Figure 3). The factors involved in the survival of sperm in SSTs have not been completely determined. However, the presence of calcium, zinc, sodium, phosphorus, sulfur, chlorine, and potassium in the SST microenvironment was associated with sperm storage [35]. The presence of lipid components [14], carbonic anhydrase [35], and aquaporins [36], as well as the expression of multiple cytoskeleton protein genes [37] in the SST cells, suggest that these components play essential physiological functions in prolonged sperm storage. In a previous study, less than 2% of an inseminated dose of 100 to 200 million spermatozoa was found in the SSTs of either turkeys or chickens [38], which might explain why even large insemination doses can result in low fertility rates [39]. The factor that affects sperm contents in SSTs the most is the insemination time, not the inseminated dose [15]. Thus, in old hens, inseminations performed frequently and with a moderate number of spermatozoa might be more efficient than inseminations performed with higher doses at longer intervals.

## 5. Conclusions

We demonstrated that the differences in fertilizing capacity between mature and old hens depended on morphological characteristics, such as the inner and outer diameter of the SSTs, epithelium height, and sperm contents in the SSTs. In old hens, sperm could not be retained within the SSTs, resulting in fertility decline. In such cases, increasing the frequency of insemination might be the method of choice to increase fertility.

## Figures and Tables

**Table 1:** Reproductive organ appearance of mature (35–39 weeks) and old (73–75 weeks) hens.

Parameters	Hen Groups		*p*-Value
	Mature	Old	
Body weight (kg)	1.8 ± 0.0 ^b^	2.1 ± 0.1 ^a^	0.03
Ovary weight (g)	36.3 ± 3.5	35.2 ± 2.1	0.53
Oviductal weight (g)	35.8 ± 3.0	38.4 ± 2.9	0.96
Oviductal length (cm)	67.1 ± 5.9	67.2 ± 2.7	0.15

^a,b^ Means with different superscript letters within a row differed significantly (*p* < 0.05).

**Table 2:** Comparison of follicular type, number, and diameter in mature (35–39 weeks) and old (73–75 weeks) hens.

Parameters			Hen Groups		*p*-Value
		Follicle Types	Mature	Old	
Quantity (Number of follicles)		SWFs (1–3 mm)	26.8 ± 1.9 ^a^	23.0 ± 1.8 ^b^	0.03
		LWFs (3–5 mm)	17.7 ± 1.9 ^a^	14.0 ± 1.7 ^b^	0.04
		SYFs (5–10 mm)	11.2 ± 2.4	8.3 ± 2.7	0.10
		LYFs (>10 mm)	2.5 ± 0.8 ^a^	1.3 ± 0.5 ^b^	0.01
		TF (1–10 mm)	55.7 ± 4.2 ^a^	45.3 ± 4.1 ^b^	0.03
Top five largest yellow follicle diameters	Major axis (mm)	F1 (mm)	36.6 ± 3.5	35.6 ± 8.8	0.76
		F2 (mm)	33.4 ± 2.5	30.0 ± 8.0	0.39
		F3 (mm)	30.0 ± 3.0	23.9 ± 9.5	0.21
		F4 (mm)	23.1 ± 3.7	16.3 ± 6.2	0.16
		F5 (mm)	16.9 ± 4.7 ^a^	10.1 ± 1.8 ^b^	0.04
	Minor axis (mm)	F1 (mm)	32.7 ± 2.5	31.1 ± 8.1	0.63
		F2 (mm)	28.9 ± 2.3	25.4 ± 7.1	0.20
		F3 (mm)	24.6 ± 1.8	20.1 ± 5.7	0.21
		F4 (mm)	20.2 ± 3.8	14.0 ± 5.7	0.16
		F5 (mm)	14.9 ± 3.8 ^a^	8.8 ± 1.8 ^b^	0.36

^a,b^ Means with different superscript letters within a row differed significantly (*p* < 0.05). SMFs—small white follicles (1–3 mm), LWFs—large white follicles (3–5 mm), SYFs—small yellow follicles (5–10 mm), LYFs—large yellow follicles (>10 mm), and TF—total follicles (1–10 mm). F1—first yellow follicle, F2—second yellow follicle, F3—third yellow follicle, F4—fourth yellow follicle, and F5—fifth yellow follicle.

**Table 3:** Structural analysis of sperm storage tubules in mature (35–39 weeks) and old (73–75 weeks) hens.

Parameters	Hen Groups		*p*-Value
	Mature	Old	
Number of folds, *n*	23.0 ± 1.1	23.8 ± 1.4	0.59
Fold length (µm)	463.6 ± 36.7	485.3 ± 42.4	0.79
SSTs epithelium height (µm)	16.3 + 1.3 ^a^	13.6 ± 2.6 ^b^	0.04
Outer diameter of the SSTs (µm)	30.6 ± 5.8 ^a^	55.5 ± 8.9 ^b^	0.04
Inner diameter of the SSTs (µm)	6.9 ± 1.7 ^a^	27.5 ± 3.7 ^b^	0.03
SSTs per fold, *n*	338.0 ± 68.6	306.7 ± 64.0	0.90

^a,b^ Means with different superscripts within a row differed significantly (*p* < 0.05).

**Table 4:** Spermatozoa (×10^4^) that were recovered from mucosa washing of the UVJ and percentage of SSTs containing sperm after artificial insemination for 24 h.

Parameters	Hen Groups		*p*-Value
	Mature	Old	
Spermatozoa (×10^4^)	143.2 ± 14.0 ^a^	114.6 ± 17.1 ^b^	0.03
SSTs containing with sperm (%)	26.8 ± 3.4 ^a^	6.8 ± 1.7 ^b^	<0.01

^a,b^ Means with different superscripts within a row differed significantly (*p* < 0.05).

## Data Availability

The data are available upon request from the corresponding author.
